# Toward a New Generation of Organic Coatings for Corrosion Protection Based on Poly(phenylene methylene)

**DOI:** 10.1021/acs.langmuir.5c02887

**Published:** 2025-10-08

**Authors:** Walter R. Caseri, Markus Niederberger

**Affiliations:** Laboratory for Multifunctional Materials, Department of Materials, 27219 ETH Zürich, Vladimir-Prelog-Weg 5, 8093 Zürich, Switzerland

## Abstract

Metal corrosion is a costly surface phenomenon that is typically prevented by organic coatings based on cross-linked resins. However, these coatings have severe limitations in recycling, and small defects are difficult to detect and repair, which can lead to hidden corrosion and catastrophic material failure. Modified poly­(phenylene methylene) (PPM) coatings overcome these shortcomings because they are thermoplastic and recyclable and can be customized by incorporating functional additives or through side chain engineering, resulting in self-healing and fluorescent coatings for straightforward, on-demand, and nondestructive quality control over the entire time range from application to service life. Furthermore, the use of PPM-based polymers might eliminate the need for surface pretreatments, which are commonly performed to improve coating adhesion. To date, however, PPM-based corrosion protection has only been validated on a single yet significant aluminum alloy (AA2024). Broadening its applicability to other metals/alloys, exploring powder coating techniques, and enhancing glass transition temperatures while retaining self-healing functionality are key directions for future research.

## Graphical abstract

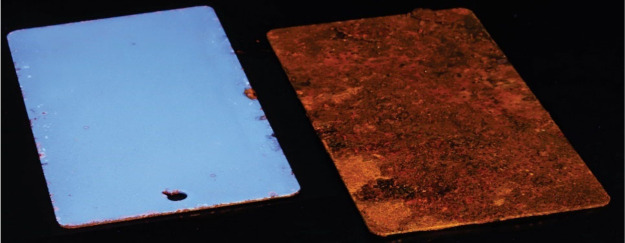

## 1 Introduction

Corrosion of metals is a dreaded surface phenomenon that is responsible for catastrophic failures of engineering components and structures. Localized corrosion is particularly insidious because it is difficult to recognize, predict and combat.^1,2^ It is caused by the attack of oxidizing substances from the environment on a small, limited area of a metal surface and often leads to the failure of technical systems.^3,4^ Therefore, metals susceptible to corrosion must be protected. Organic coatings account for two-thirds of all annual spending on corrosion protection.^5^

Protective organic coatings are single- or multilayer systems designed to physically separate surfaces from oxidizing substances in the environment.^6^ Typically, such coatings are complex formulations consisting mainly of a polymer-resin-based binder, pigments, and other ingredients.^7,8^ Currently, polymers from the class of duromers, such as epoxy resins or alkyd resins, are most commonly used for organic coatings.^8–10^ These polymers are highly cross-linked and are usually produced *in situ* by mixing two components.^8^ They are valued, for example, for their chemical and physical stability and their temperature resistance. However, these polymers do not melt and are generally insoluble, which hampers their processability. As a result, the corresponding coatings are difficult to separate from the adherent metals at the end of their life cycle, which makes it difficult to recover the materials.^11,12^ In addition, surface defects impair the effectiveness of such coatings, as corrosive substances from the environment can slowly penetrate the underlying metal surfaces, causing local corrosion and blistering that can spread and lead to unexpected material failure.^8^ Due to the cross-linked structure of those coatings, such defects usually persist.

To improve the adhesion between the coatings and the metal, pretreatment of the metal surfaces is often necessary, such as cleaning procedures or surface modifications.^13,14^ In this context, surface treatments based on silane and chromate chemistry are often used to improve corrosion resistance.^15,16^ However, chromate-based chemicals are toxic and carcinogenic, leading to increasingly stringent legal regulations.^17,18^ Therefore, intensive research is being conducted to find new coating applications that eliminate the need for this pretreatment.^15,16^

Major advances in addressing these limitations have been made recently using poly­(phenylene methylene)-based polymers, which combine self-healing capabilities, compatibility with conventional processing, and easy removal for recycling. They eliminate the need for hazardous surface pretreatments and enable early detection of coating defects and localized corrosion at metal surfaces. Coatings with such multifunctional properties are poised to define the next generation of corrosion protection, with wide-ranging impact across diverse technological sectors and industries.

## 2 A New Generation of Corrosion Protective Coatings

### 2.1 A Widely Forgotten Multifunctional Polymer

Poly­(phenylene methylene) (PPM) (Figure 1a) was first synthesized in the 19th century.^19^ But despite its early discovery it has received limited attention, with only sporadic reports appearing in the literature over the following decades.^20–25^ Although rediscovered by chance, poly­(phenylene methylene) has the potential to “open new horizons for the entire science community”.^26^ Our research group identified PPM as a byproduct during the synthesis of tungsten oxide nanowires in benzyl alcohol.^27^ In fact, the observation of PPM formation during the synthesis of metal oxide nanoparticles in benzyl alcohol is quite common^28,29^ and may affect the optical properties of the inorganic product. Given the difficulty of removing PPM from nanoparticles, it is crucial to modify the synthesis conditions to suppress its formation.^30^ In any case, our observation prompted a targeted investigation of the polymer, which led to significant progress in its synthesis. We established a scalable, one-step catalytic polymerization process using benzyl chloride, enabling the reproducible synthesis of PPM in quantities of more than 100 g on a laboratory scale.^31^ More recently, attention has been paid to the unexpected fluorescence of PPM upon irradiation with UV light.^32,33^ Evidence was found that this property is likely a result of a rare phenomenon called homoconjugation, which arises by overlap of p-orbitals of phenylene moieties although they are separated by methylene groups, as illustrated in Figure 1b. The characteristic blue light, which is emitted after excitation, is predestined for the detection of cracks and other small defects in films of PPM, as outlined in more detail below.

**Figure 1:**
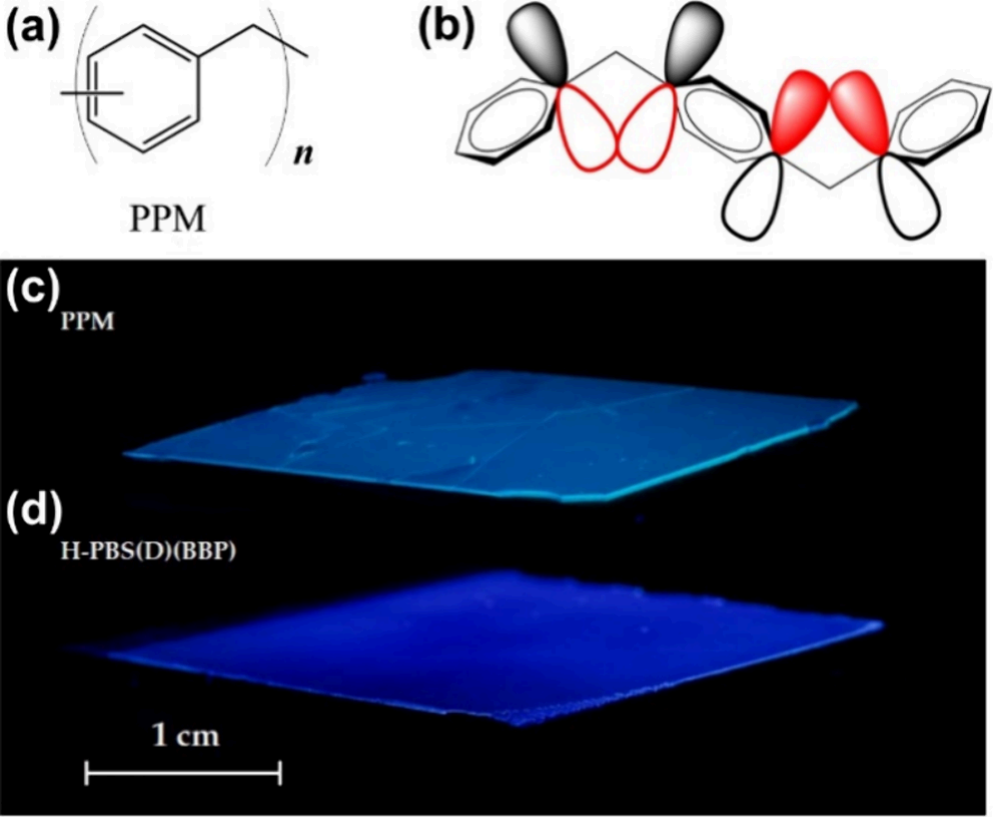
(a) Molecular structure of poly­(phenylene methylene) (PPM), which is composed of aromatic units separated by methylene groups. (b) Schematic representation of homoconjugation in PPM: p orbitals of phenylene rings overlap even if they are electronically separated by a methylene group. (a) and (b) reproduced from ref 39. CC BY 4.0. (c) Photograph of a PPM coating prepared by hot-pressing on the aluminum alloy AA2024 revealing the formation of cracks. (d) Photograph of a crack-free PPM coating containing additives after 2 weeks. The pictures were taken under irradiation with UV light, causing the characteristic blue fluorescence of PPM. H-PBS­(D) designates a hybrid-like mixture of PPM and a polycondensation product of benzyltriethoxysilane (PBS) prepared from dispersion. (c) and (d) Reproduced from ref 34. CC BY 4.0.

In addition to its fluorescence, PPM exhibits a number of remarkable materials properties: hydrophobicity,^20,34,35^ high thermal stability (decomposition commencing at 450–470 °C),^20–22,31^ resistance toward oxidizing agents,^34^ good barrier properties,^20^ and thermoplastic processability.^32,34^ This unique combination of properties makes PPM an attractive candidate for coating applications, and indeed its potential in this area was suggested some time ago.^35–38^ However, to the best of our knowledge, its practical suitability has only recently been evaluated experimentally. These studies revealed that pure PPM coatings are inherently brittle and prone to crack formation (Figure 1c),^34^ which currently limits their viability for corrosion protection. Nonetheless, Figure 1c also highlights a key advantage: the inherent fluorescence of PPM enables sensitive and straightforward visual detection of microcracks and defects that would otherwise be difficult to identify under ambient lighting conditions—an issue common in transparent polymeric coatings.

### 2.2 Strategies to Improve Coatings Based on Poly­(phenylene methylene) (PPM)

To improve the quality of PPM-based coatings, two strategies were developed and experimentally validated: (i) blending PPM with additives^34^ and (ii) modification of the PPM main chain with side groups (i.e., side-group engineering).^40^ In all of the cases described below, aluminum alloy AA2024 served as the substrate of choice. AA2024 is known for its high strength and widespread use in aerospace applications. However, it is also particularly susceptible to localized corrosion, especially in chloride-rich environments, making it an ideal candidate for evaluating corrosion protection strategies.^15^ In engineering applications, chromate treatment of AA2024 surfaces is typically required to ensure reliable performance of the coatings. In the case of PPM, this toxic process can be replaced. The AA2024 possesses hydroxyl groups on its surface,^41^ which can be used for chemical reactions with trialkoxysilanes^42,43^ to improve the compatibility of the apolar PPM with the highly polar aluminum surface. Specifically, benzyl triethoxysilane was employed, because the chemical nature of benzyl groups is close to that of the constitutional repeat units of PPM. Without such surface pretreatment, PPM films tend to detach from the surface and form more cracks within seconds of applying neat PPM.^34^ Corrosion tests were performed by exposing coated AA2024 to a simulated marine environment, i.e. to a 0.6 M (3.5% w/v) NaCl solution. Chloride ions are mainly responsible for the localized corrosion of passive metals.^44,45^

#### 2.2.1 Additives

The quality of PPM coatings can be significantly improved by additives.^34^ This was demonstrated with films of about 40 μm thickness, which were simply prepared by hot pressing PPM powders. To reduce cracks and bubbles in the coatings, benzyl butyl phthalate (BBP) was introduced as a plasticizer (17% m/m). This addition resulted in a reduction in the glass transition temperature of PPM from 65 to 35 °C, indicating that the mobility of the polymer chains increased. Moreover, polybenzylsiloxanes (1% m/m) enhanced the viscosity of PPM in the molten state (as present in the hot press) and further improved the quality of the films. It is well established that higher viscosity helps reduce bubble and crack formation.^7,46,47^ As a result, uniform films were obtained that remained intact and free of cracks for several months (Figure 1d).

The surface properties of the coatings were characterized through contact angle measurements, revealing their hydrophobic nature. Advancing water contact angles (θ_a_) of 105° and receding angles (θ_r_) of 98° were measured, indicating strong water repellency. This is a desirable feature for corrosion resistance in aqueous environments. Besides, the rather low contact angle hysteresis (7°) indicates that the surfaces of the coatings are quite homogeneous.

The resistance of the coatings to pitting corrosion was investigated by cyclic potentiodynamic scans, followed by potentiostatic analyses. The results revealed a pronounced barrier effect provided by the coatings. This feature is pivotal as the ability to isolate the metal surface from the corrosive environment is decisive. Accordingly, intact coatings offered effective corrosion protection. For instance, potentiostatic analyses conducted at a constant potential (approximately −0.3 V vs SCE, as determined from prior anodic polarization measurements) indicated effective protection over the entire 28-h stress test (Figure 2a), placing these coatings among the most effective studied using this method. Most notably, however, the graph in Figure 2a displays a very sharp increase in electric current after approximately 5.5 h, which quickly returned to its initial low level and remained stable until the end of the measurement. Obviously, a metastable pit formed, but it was suppressed rapidly (within a few minutes), demonstrating the ability of the coating to compensate for and mitigate defects under pit attacks without external stimulus. This observation is in line with the fact that the samples showed no visible signs of corrosion when observed with a microscope at the end of the potentiostatic experiments (Figure 2b).

**Figure 2:**
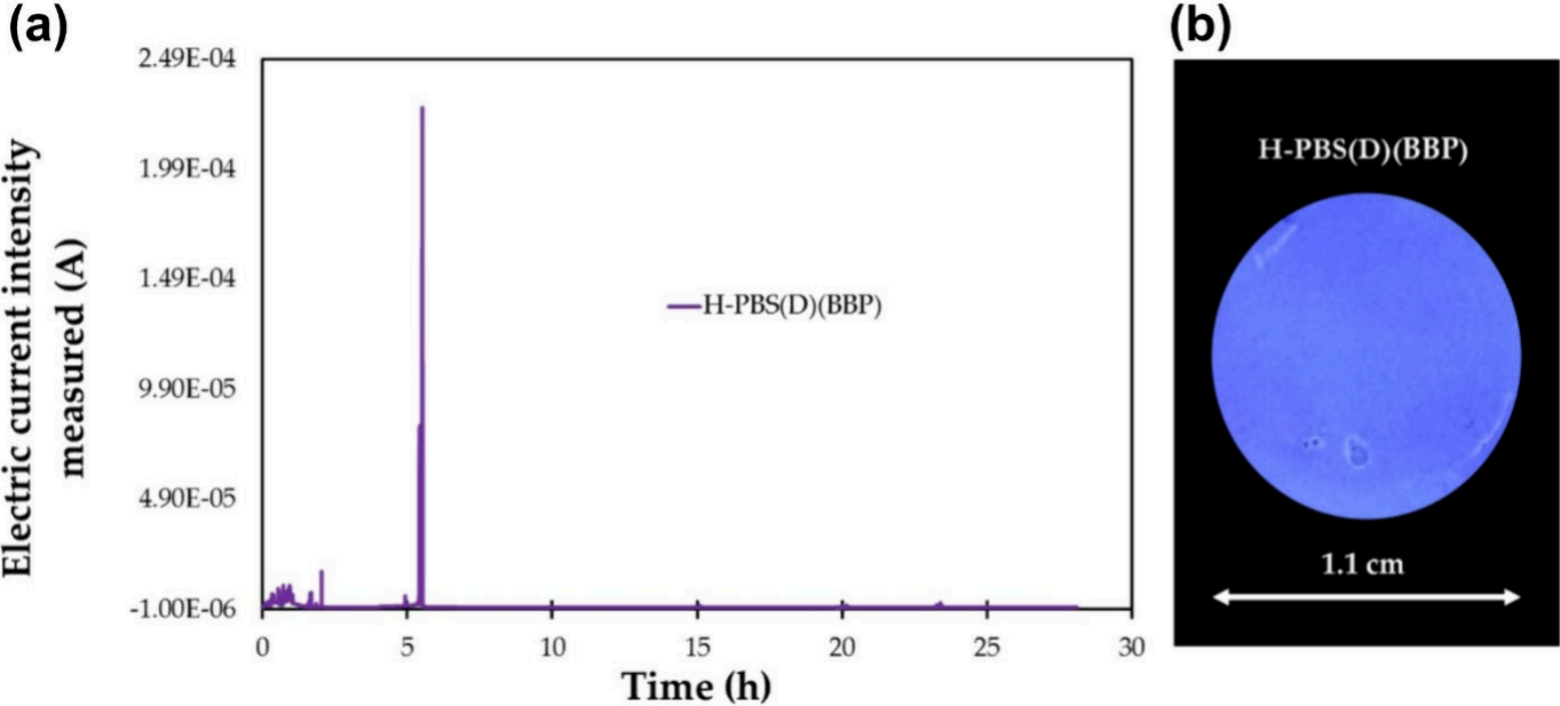
(a) Potentiostatic scan at a constant potential of about −0.3 V vs SCE of a coating of PPM with additives (see text) on the aluminum alloy AA2024 (relevant in aerospace industry) exposed to 0.6 M NaCl solution. The peak after ca. 5.5 h is due to a defect in the coating which emerged and subsequently vanished rapidly. (b) State of the surface of the coating after the scan, i.e., after exposure to the corrosion medium for 28 h (picture taken at irradiation of 365 nm light, causing the characteristic blue fluorescence of PPM). The image reveals that the coating is free of visible defects. Reproduced from ref 34. CC BY 4.0.

Despite this success, it is important to consider the potential issue of plasticizer migration. Over periods ranging from years to decades, plasticizer molecules like BBP can gradually migrate and accumulate on the polymer surface or at the polymer|substrate interface. This migration can not only shorten the lifetime of the coatings, but also pose environmental risks as the migrated plasticizers can eventually be released into the environment.

#### 2.2.2 Side Group Engineering

In general, the chemical and physical properties of polymers can be modified by the copolymerization of two or more monomers. For PPM, this approach enables the introduction of specific side groups into the main chain simply by copolymerization of benzyl chloride and substituted benzyl chlorides in the desired ratio (side group engineering). Side groups can reduce the interactions between the polymer chains, which promotes the sliding of the main chains.^48^ In this way, side group engineering was applied to reduce crack formation of PPM-based coatings without addition of a plasticizer.^39,40^ Importantly, the characteristic blue fluorescence of PPM was fully preserved in the copolymers. The onset of decomposition of the copolymers in air also remained high (at least 380 °C according to thermogravimetric analyses). Copolymers with about 13% mol/mol octyloxy side chains (based on the total number of phenylene methylene units in the copolymer) proved ideal for forming high-quality films (Figure 3a).^39^ Films with a thickness of about 30 μm were prepared by hot pressing. The very small contact angle hysteresis of 1° (θ_a_ and θ_r_ 102° and 101°, respectively) indicates a homogeneous surface.

**Figure 3:**
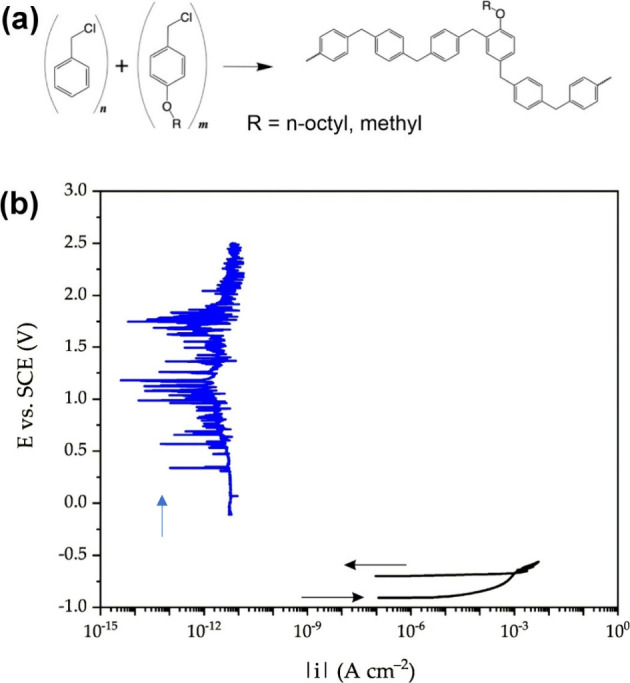
(a) Reaction scheme for the preparation of copolymers by side group engineering, R = methyl or octyl. This method allows for a wide range of copolymers as many side groups are available. Reproduced from ref 39. CC BY 4.0. (b) Representative potentiodynamic anodic polarization curves obtained for blank AA2024 (black; cyclic polarization) and for a copolymer with 9.0% methoxy groups related to the total amount of phenylene units (blue) in naturally aerated 0.6 M NaCl solution (frequently considered as artificial marine environment). Potential scan rate: 10 mV/min. Adapted from ref 40. CC BY 4.0.

Anodic polarization provides insight into the corrosion behavior of metals/alloys and the protective effect of organic coatings on metallic substrates. In the presence of chloride ions, or generally corrosion-triggering ions, this test also assesses the effects of such species on the metal behavior and the barrier properties of the coatings against the diffusion of these charged species. The anodic polarization curves of the samples with octyloxy groups were strongly affected by the coating. In the case of bare aluminum alloy AA2024, even slightly anodic overpotential values resulted in an abrupt increase in current density, confirming the absence of any passivation regime. The coated samples exhibited quite low passivation-like current densities, suggesting that the copolymer coating protected the aluminum alloy surface very efficiently from the electrolyte solution. Experiments using accelerated cyclic electrochemical technique (ACET), a standard test for evaluating the quality of industrial paints and coatings, which was applied to coatings with an artificial scratch, revealed self-healing of the damaged coating within 3 h.^49^ A tentative explanation for the experimental observation is that the exothermic aluminum dissolution locally increased the temperature of the coating around the scratch above its relatively low glass transition temperature, thereby accelerating the mobility of the polymer chains, which moved to heal the discontinuity in the polymer layer. On the other hand, further anodic polarization tests showed that corrosion protection was maintained for about 12 h,^39^ after which the barrier effect of the coating gradually decreased. It seems that defects slowly formed, probably due to the penetration of the aqueous electrolyte through pores in the films, and that small metastable depressions formed at the interface between the metal and the solution beneath the coating layer, which could trigger delamination over an increasing area. This may have been facilitated by the low glass transition temperature (31 °C) of the octyl derivative, which could reduce the corrosion protection effect of the coatings even at temperatures just above room temperature.

To increase the glass transition temperature, the length of the pendant alkyl groups can generally be reduced.^48,50^ Accordingly, the shortest alkyl group, i.e., the methyl group, was selected.^40^ A methoxy group fraction of 9.0% mol/mol in the copolymer proved to be effective for corrosion protection. The glass transition temperature (*T*_g_) of this copolymer indeed exceeded that of the copolymer with the octyloxy groups (approximately +30 °C) and reached about 65 °C. Once again, films with thickness of about 30 μm were produced by hot pressing on surface-modified aluminum AA2024.

The coating significantly expanded the stability window of the aluminum alloy AA2024 in anodic polarization tests and ensured a very low and stable current density across the entire potential range investigated, i.e., approximately +2.5 V anodic overpotentials (Figure 3b). This indicates a compact and almost defect-free coating that effectively blocks the diffusion of water and the migration of chloride ions from the solution to the underlying metal surface. It is noteworthy that the coating with methoxy groups remained completely intact after the corrosion test when examined under an optical microscope. Compared to PPM with additives or the copolymer with octyloxy side groups, the methoxy-substituted copolymer coating showed superior performance, exhibiting lower current density under potentiodynamic anodic polarization.

To assess whether the copolymer with methoxy groups can still inhibit corrosion at defective sites despite its higher *T*_g_, artificial holes (diameter approximately 0.5 mm) were introduced into the coatings. Electrochemical impedance spectra were recorded during 39 h of immersion to track the evolution of the coated system. Representative Bode modulus spectra are shown in Figure 4a, together with the corresponding impedance modulus at a lower frequency of 0.01 Hz (|*Z*_0.01 Hz_|), which accounts for the coating resistance and thus its integrity (Figure 4b). The impedance increased significantly between 5 and 8 h and then remained above 10^8^ Ω cm^2^ for up to 60 days (i.e., until the end of the experiment) of exposure to the chloride-containing solution. It therefore appears that after 7–8 h, sustainable corrosion protection was restored, although further experiments with accelerated anodic aging suggested that the durability of the repaired coating may not have been fully restored. Nevertheless, the coatings preserve their ability to stop localized corrosion by a self-healing process. This is confirmed by visual observation with a microscope (Figure 4c). The initial artificial hole is represented by the bright areas under visible light caused by corrosion products. These corrosion products are covered again with copolymer as evident from the image taken under UV-light irradiation. It appears from the structure of the film covering the damaged site that the polymer flowed into the artificial hole under the action of local heating. Indeed, self-healing of damaged coatings can be triggered by exothermal reactions resulting from corrosion.^51,52^ Although the glass transition temperature of the copolymer is about 40 °C above room temperature, the heat released during the corrosion reaction could render the copolymer flowable, nonetheless.

**Figure 4:**
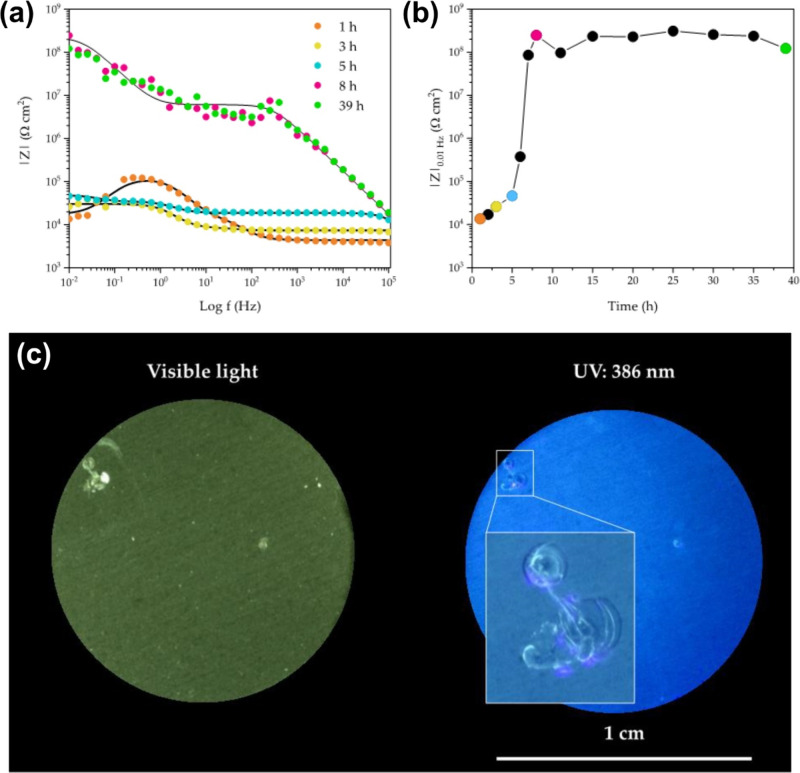
(a) Representative Bode impedance spectra of a coating of a PPM copolymer substituted with 9.0% methoxy groups with an artificial scratch upon exposure to 0.6 M NaCl solution. Substrate: aluminum alloy AA2024. Dots represent experimental data (frequencies) and the lines represent fitted spectra by equivalent electric circuit model. (b) Evolution of |*Z*|_0.01 Hz_ over exposure time; the colors correspond to the times indicated in (a), showing that the surface exposed to the corrosive medium by an artificial scratch in the coating was efficiently protected again after about 7–8 h. (c) Optical microscopy images of the surface of damaged PPM copolymer coatings with 9.0% methoxy groups exposed for 60 days to 0.6 M NaCl solution. The blurred area represents the repair of a damage in the coating. Reproduced from ref 40. CC BY 4.0.

The two examples of substituted PPM highlight how side-group engineering can be a powerful approach to designing superior coatings for corrosion protection. Given the simplicity of the copolymerization process and the wide availability or easy synthesis of many comonomers, this strategy offers enormous potential for customizing coating properties for specific performance requirements.

## 3 Moving Forward

### 3.1 Technological Aspects

PPM-based coatings are characterized by their excellent corrosion protection properties. They are distinguished by a remarkable ability of self-healing and easy detection of defects through fluorescence. Their solubility in organic solvents also facilitates recycling, making them an eco-friendly option. These attributes position PPM-based materials as promising contenders for the next generation of advanced, high-performance, and sustainable coating systems. However, while these coatings offer many benefits, there is still room for further improvement in their technical performance, as discussed below.

- Surface pretreatment: The coating process could become more economical and environmentally sustainable by eliminating the need for pretreatment of the metal surface.
- Adhesion: No particular efforts have been made to improve the adhesion of PPM-based coatings to the substrate. This challenge can be addressed by using specific additives or by exploiting side-group engineering to equip the copolymer with anchoring groups. Blending the polymers with compounds that interact with both the substrate and the polymer matrix (i.e., linkers) may be a simple and effective solution. For example, amphiphilic additives with a polar group that binds to the aluminum surface and a less polar segment that is compatible with the polymer could act as internal surface modifiers. However, a potential disadvantage of this approach is that excess additives would remain in the coating, which could compromise long-term corrosion resistance. In particular, the possibility of additive migration over long periods of time must be carefully evaluated. Alternatively, side group engineering can improve direct adhesion to the metal surface by using covalently bound anchoring groups. The incorporation of side groups with a strong affinity for the substrate can eliminate the need for separate surface treatment. For example, carboxylate or carboxylic acid groups can interact with metal surfaces and form metal carboxylate bonds, establishing strong chemical links between the polymer and the substrate. In addition, alcohol or alkoxide groups can coordinate with metal atoms on the surface, promoting the adhesion of the coating to the substrate.
- Substrates: To date, PPM-based coatings have only been tested on the technically relevant aluminum alloy AA2024. This alloy is particularly susceptible to corrosion and is therefore well suited for fundamental corrosion studies. However, it can be assumed that the effectiveness of PPM-based coatings is not limited to AA2024, but applies to a wide range of metals, including various types of steel. Although there is no reason to expect failure with coating-specific metallic substrates, extending the experimental investigations to other metals will be an important step forward.
- Deposition: Coatings have so far been produced by hot pressing or spray coating. These processes deliver uniform, high-quality films, but hot pressing is limited to flat surfaces, and spray coating has so far relied on organic solvents. Although such solvents are still widely used in coating technology, they raise both environmental and economic concerns. By switching to spray coating techniques that use aqueous dispersions, the need for organic solvents could be significantly reduced or even eliminated completely. Alternatively, powder coating, a widely used industrial process, could also be suitable for PPM-derived polymers. This solvent-free process allows excess powder to be recovered and reused, minimizing waste, reducing costs, and lowering environmental impact.
- Molar mass: The influence of molecular weight on the corrosion protection effect is an aspect that has not yet been investigated. As a rule, high molar masses increase the glass transition temperatures up to a certain plateau value and can also help to reduce the formation of cracks. A simple method has recently been developed to increase the molecular weights of PPM-based polymers through a low degree of branching,^53^ which could also be investigated with regard to further reducing the layer thickness required to protect metal substrates from corrosion.

### 3.2 Sustainability

While recyclability of the polymer makes PPM-based materials promising in terms of the reduction of the end-of-life impact and of the employment of virgin feedstock materials, they are petrol-derived materials. To further enhance sustainability, exploring naturally sourced monomers for the phenylmethylene core represents a critical next step. An ecotoxicity assessment of PPM-based polymers is still lacking. Acute and chronic tests with recognized biomarkers (e.g., *Daphnia magna*, small crustaceans) could provide insight here. Life cycle assessment (LCA) is a valuable approach for quantitatively estimating the environmental impact of processes and materials. Corresponding studies on PPM are already underway.

## 4 Conclusions

In summary, PPM-based coatings represent a promising alternative to conventional cross-linked resins commonly used for corrosion protection of metals. These innovative coatings offer a unique combination of processability with techniques commonly used for thermoplastic polymers, intrinsic self-healing capabilities, fluorescence-based defect detection, and recyclability. Moreover, they can be produced in large quantities through simple one-step-reactions. Side-chain engineering allows the development of polymers with tailored properties for specific applications. Future developments could further improve their performance by enabling precise control of the glass transition temperature, eliminating the need for surface pretreatment, and supporting solvent-free application methods. Moreover, expanding their applicability to a wide range of metal substrates will be key to establishing PPM-based materials as a new generation of versatile, sustainable, and high-performance protective coatings.
